# Altered semantic prediction error processing with increasing schizotypal and autistic traits

**DOI:** 10.1038/s41398-026-04479-4

**Published:** 2026-09-30

**Authors:** Elisabeth F. Sterner, Verena F. Demler, Rachel Lenz, Lucy J. MacGregor, Christoph Mathys, Franziska Knolle

**Affiliations:** 1 School of Medicine and Health, Department of Diagnostic and Interventional Neuroradiology, Technical University of Munich, Munich, Germany; 2 School of Medicine and Health, TUM-NIC Neuroimaging Center, Technical University of Munich, Munich, Germany; 3 Department of Experimental Psychology, Ludwig-Maximilians University Munich, Munich, Germany; 4 School of Medicine and Health, Department of Anesthesiology and Intensive Care, Technical University of Munich, Munich, Germany; 5 MRC Cognition and Brain Sciences Unit, University of Cambridge, Cambridge, UK; 6 Interacting Minds Centre, Aarhus University, Aarhus, Denmark

**Keywords:** Psychiatric disorders, Human behaviour

## Abstract

Predictive processing has been proposed as a framework for symptom development in autism (ASD) and schizophrenia (SSD) spectrum disorders, with ASD being associated with an overweighting of (low-level) sensory evidence whereas SSD is characterized by an overweighting of (high-level) prior beliefs, both resulting in altered prediction error processing. This study investigated these hypotheses and their electrophysiological correlates in subclinical expressions of ASD and SSD during language processing. Participants completed an auditory comprehension task that manipulated the precision of high-level semantic prior beliefs and low-level sensory evidence. Hierarchical Bayesian belief-updating modeling and electroencephalography were used to examine whether imbalances in the weighting of prior beliefs and sensory evidence were reflected in altered processing of semantic prediction errors, characterized by mean N400 amplitudes. Computational modeling revealed that increasing schizotypal traits were associated with significant overweighting of prior beliefs, while autistic traits did not show a significant shift. Linear mixed-effect models of mean N400 amplitudes indicated that schizotypy-related overweighting of semantic prior beliefs was reflected in a reduced semantic prediction error signal, indexed by smaller N400 differences between high predictability sentences and both low predictability and high predictability mismatch sentences. A similar but weaker and less distinct pattern emerged for increasing autistic traits. These findings provide converging computational and electrophysiological support for overweighting of semantic prior beliefs with increasing subclinical schizotypy. In contrast, increasing autistic traits were not associated with overweighting of sensory evidence, with electrophysiological results instead suggesting subtle alterations in the weighting of semantic prior beliefs.

## Introduction

Schizophrenia (SSD) and autism spectrum disorder (ASD) are distinct psychiatric diagnoses which share genetic predisposition, environmental risk factors, and symptom features [1, 2]. This overlap is supported epidemiologically [2, 3] and also reflected in a strong correlation of schizotypal and autistic personality traits in the general population [4, 5] where similar symptom profiles can be observed as in clinical cohorts but on a subclinical level. Contrary to this perspective, recent predictive processing accounts have suggested that diametrically opposed computational alterations give rise to symptom development in SSD [6–8] and ASD [9–11].

**Fig. 1:**
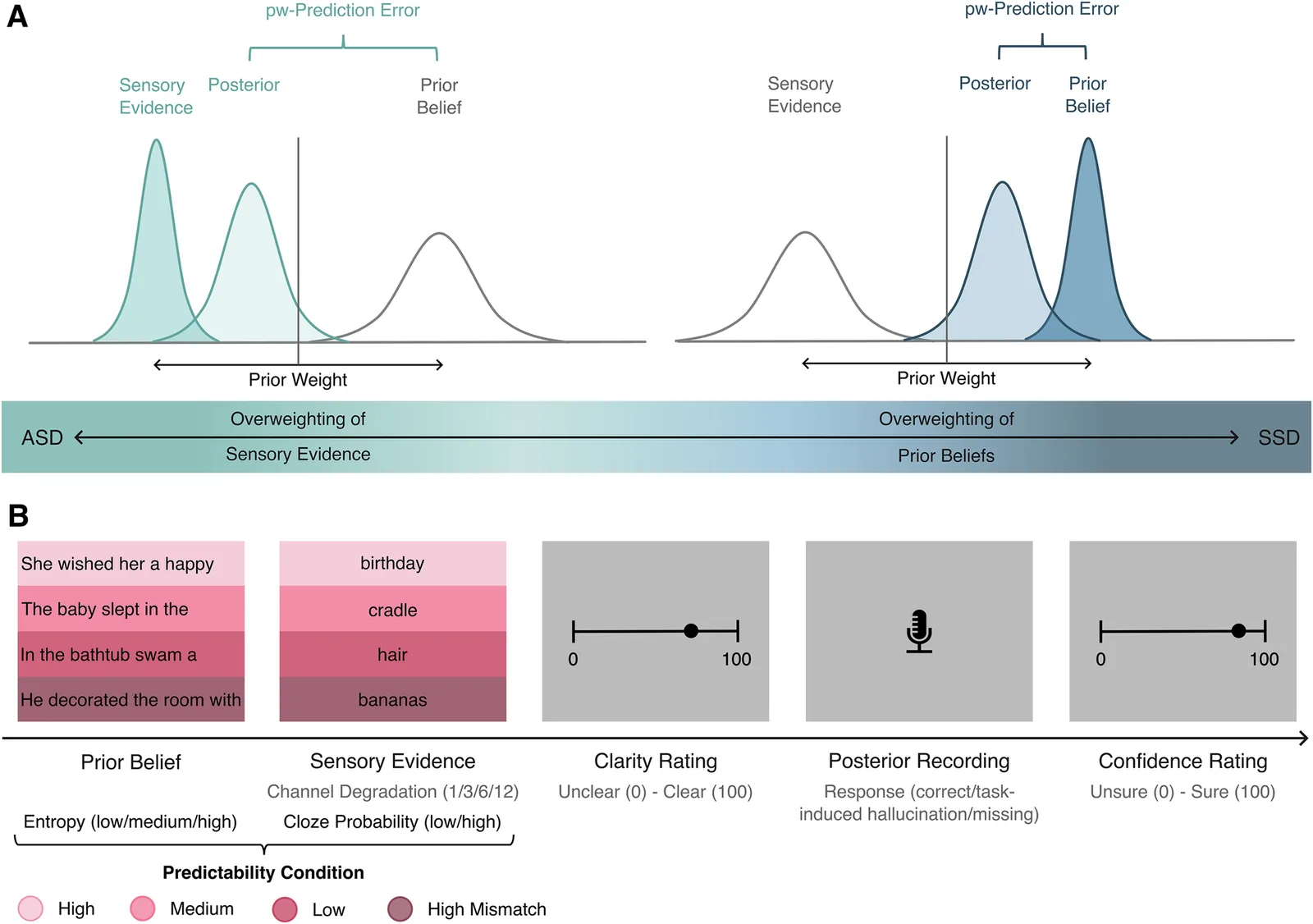
Hypotheses for ASD and SSD along the Predictive Preprocessing Continuum  and Predictive Language Processing Task. **A** ASD and SSD Mapped onto the Predictive Processing Continuum. Left shows an overweighting of sensory evidence, resulting in increased prediction error signaling (visualized as the difference between the prior beliefs and the posterior) as suggested in ASD, whereas right displays an overweighting of prior beliefs with attenuated prediction error signaling as suggested in SSD. In both cases the posterior moves towards the more strongly weighted component indicated by higher precision, i.e., lower variance of the distribution. **B** Predictive Language Processing Task. On each trial, participants listened to sentences via headphones. High-level semantic prior beliefs with varying precisions were induced by sentence beginnings with three predictability levels measured in entropy (high predictability, N = 80; medium predictability, N = 80; low predictability, N = 40). Half of the high predictability sentences (N = 40) were completed by a sentence-final word with a cloze probability of 0, forming the high predictability mismatch condition. The remaining 160 sentences were completed with the highest cloze probability response as assessed in an independent sample. This resulted in four predictability conditions (from light to dark pink): high, medium, low, and high mismatch. The precision of the sensory evidence was further modulated by the auditory clarity of the sentence-final words, which were presented in four different clarity levels (using noise vocoding with 1, 3, 6, 12 channels ranging from unintelligible to highly intelligible). After the presentation of the final word, participants rated the clarity of the target word (Likert scale: 0–100). Participants were then asked to say the word they heard and rate how confident they were with their response (Likert scale: 0–100).

Predictive processing suggests that the brain performs Bayesian inference to respond efficiently to its environment by integrating high-level prior beliefs generated from an internal model of the world with incoming low-level sensory information [12] which are weighted according to their respective precisions (i.e., inverse variances). Signals with higher precision have a greater impact on the posterior belief – the perception. The discrepancy between prior beliefs and sensory evidence, the prediction error, drives updates of the internal model (Fig. 1A) to generate more accurate prior beliefs in the future.

In ASD, low-level sensory information appears to be overweighted [13–15], giving rise to prediction errors that are disproportionately driven by misassigned sensory precision and salience [16–19], and may contribute to the feeling of sensory overload (Fig. 1A). Persistent prediction errors may propagate up the hierarchical system, leading to increased uncertainty at higher levels, which may in turn explain difficulties in forming generalizable representations in ASD [9, 10]. The reduced influence of prior beliefs [20–24] may also contribute to social and communicative difficulties, such as a tendency toward a literal language comprehension or behavioral inflexibility which may function as compensatory strategies [11].

Like ASD, SSD has been associated with a relative overweighting of low-level sensory information [25–28] and abnormal prediction error signaling [29–31], which may give rise to delusional beliefs [32]. In contrast to ASD, SSD is additionally characterized by an increased weighting of high-level prior beliefs [33–38] (Fig. 1A). This imbalance may account for the persistence of delusions and their resistance to contradictory evidence, ultimately resulting in a fixed world model. Moreover, overweighted prior beliefs may override incoming sensory input, promoting hallucinations.

These distinct profiles have led to the proposal that ASD and SSD take opposite ends along the predictive processing axis (hereafter referred to as the ASD-SSD predictive processing continuum), where ASD is defined by a relative overweighting of (low-level) sensory information and SSD by a relative overweighting of (high-level) prior beliefs (Fig. 1A) [11, 39]. However, few studies have tested this framework, computationally [13, 21] or with imaging methods [14] in shared cohorts.

The present study aims to address these limitations by focusing on semantic language processing, a core impairment in both conditions [40, 41], which is also linked to broader phenomenology, including verbal hallucinations in SSD or communication difficulties in ASD. Language processing also provides a valuable framework for studying predictive processing mechanisms as it requires dynamic weighting of prior knowledge (i.e., contextual information) with sensory input (i.e., the speech signal) [42, 43] to allow for accurate comprehension even under challenging conditions (i.e., noisy environments). When this process is disrupted – either due to an overweighting of incoming sensory information, as suggested in ASD, or an overweighting of high-level prior beliefs, as proposed in SSD – language processing may become biased, resulting in misperceptions. Building on this approach, we previously designed an auditory predictive language task in which we manipulated the strength of semantic prior beliefs by presenting sentences with varying levels of predictability, and varied the precision of sensory evidence, by altering auditory clarity and surprisal of sentence-final words. Using a Bayesian belief updating model, we found that in two independent samples (N_1_ = 109, N_2_ = 55) higher schizotypal traits were associated with an overweighting of semantic prior beliefs and a higher frequency of misperceptions [35].

**In this study, we aim to extend these findings by comparing potential predictive language processing impairments in individuals with varying levels of schizotypal and autistic traits, and importantly, investigate how potential computational alterations underlying these impairments manifest at a neurophysiological level**.

Therefore, we used electroencephalography (EEG), focusing on the N400 component, an event-related potential (ERP) which is sensitive to contextual predictability, such that less predictable words elicit more negative amplitudes than highly predictable words [44]. Within the predictive processing framework, the N400 is operationalized as a lexico-semantic prediction error signal that emerges when meaning is inferred from an incoming speech signal [45, 46]. Following the predictive processing accounts of SSD and ASD, we expected distinct patterns of computational alterations and corresponding N400 modulations for autistic and schizotypal traits.

We hypothesized that individuals with increasing autistic traits would overweight sensory evidence, leading to heightened sensitivity to unexpected input manifested as a larger N400 for unpredictable compared to predictable sentence endings (Fig. 1A). However, previous research only partially supports this hypothesis as multiple studies report intact N400 amplitude magnitudes [47–51] or attenuated N400 effects, including children [52, 53] and adults [54, 55].

For increasing schizotypal traits, we hypothesized that individuals would demonstrate overweighting of high-level semantic prior beliefs. As this may reduce sensitivity to unexpected input, we expected smaller N400 amplitude differences, indicating an attenuated prediction error signal (Fig. 1A). Supporting this, previous studies have repeatedly found a reduced N400 effect between semantically matching and mismatching word pairs across different disease stages, from subclinical schizotypal traits [56] to clinical high risk [57], and clinical stages [58].

While we were mainly interested in the N400 time window, previous studies using sentence processing paradigms also associated late positive components, such as the P600, with semantic processing [59–61]. We therefore also performed a control analysis during a later time window.

Taken together, by investigating these effects in the general population, the present study aims to mechanistically test the ASD-SSD predictive processing continuum hypothesis and provide a more nuanced computational and neurophysiological understanding of potential language processing deficits beyond clinical manifestations.

## Methods

### Participants

**Fig. 2:**
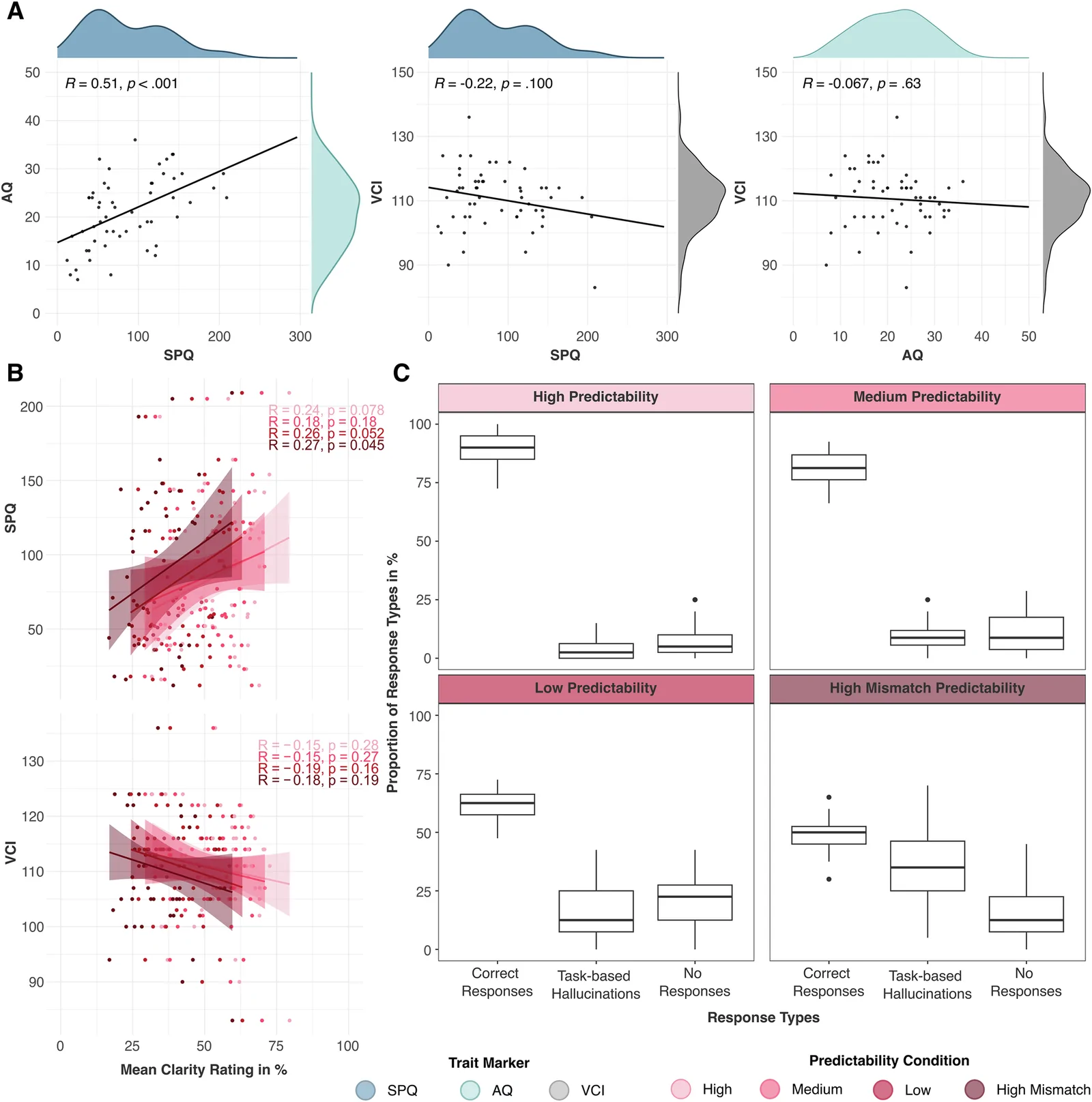
Subclinical, Cognitive Traits and Task Behavior. **A** Association between subclinical and cognitive trait markers. Autistic (AQ) and schizotypal traits (SPQ) were significantly correlated. Neither subclinical trait measure was significantly related to increased or decreased verbal comprehension ability. **B** Association between subclinical and schizotypal traits and subjective clarity ratings. Higher levels of schizotypy showed a trend toward higher clarity ratings in low, medium and high mismatch predictability conditions. Increasing verbal comprehension abilities showed a descriptively opposite, non-significant pattern. **C** Behavioral response types split by entropy conditions. Correct responses decreased with decreasing predictability manipulations while task-based hallucinations increased. No responses stayed relatively stable.

Our sample included 55 right-handed native German speakers (28 females) with normal hearing, aged between 18 and 35 (*M* = 23.73, *SD* = 3.85). All participants completed a German 5-point Likert-scale version of the Schizotypal Personality Questionnaire (SPQ; [62, 63]) to assess schizotypal personality traits (*M* = 86.56, *SD* = 50.13), and the Autism Spectrum Quotient (AQ; [64]) to measure autistic traits (*M* = 21.09, *SD* = 7.27). Additionally, we administered the subtests Vocabulary, Information, Similarities, and Comprehension of the Wechsler Adult Intelligence Scale IV (WAIS-IV; [65]) to obtain the verbal comprehension index (VCI) – a measure of verbal knowledge and reasoning abilities (*M* = 110.56, *SD* = 9.30). Figure 2 shows the distribution of the subclinical and cognitive trait markers and their correlations. A subset of the participants (N = 53) received ^1^H-MRS in a separate experimental session [35, 66–68]. All participants gave informed written consent and received financial compensation or psychology course credits after completing the experiment. The study was approved by the Ethics Commission of the Technical University of Munich (approval number 2023/270 S-KH).

### Stimuli

The task was designed to investigate predictive language processing within the predictive processing framework (Fig. 1B). The paradigm independently modulates the precision of high-level semantic prior beliefs and low-level sensory evidence. The semantic prior was formed by the presented German sentence up to the sentence-final word. The semantic prior’s precision was modulated through Shannon entropy [69] which quantifies the uncertainty about the remainder of the sentence. 40 high entropy sentences, 80 medium entropy sentences, and 80 low entropy sentences were selected, normed in an independent sample of native speakers German participants (in part drawn from [70], for psycholinguistic properties see Table S1). For the medium and high entropy conditions, the word with the highest cloze probability was selected as the sentence-final word, forming the medium and low predictability conditions. For the low entropy sentences, half of the sentences were completed by the word with the highest cloze probability, forming the high predictability condition, whereas the other half was completed by a syntactically plausible word with a cloze probability of 0, forming the high mismatch predictability condition (for examples see Fig. 1B). The precision of the sensory evidence was modulated through different levels of noise vocoding on the sentence-final word. Each word was degraded using 1, 3, 6 or 12 vocoder channels [71]. Here, perceptual clarity is increasing with rising channel numbers [72]. All sentence-final words were vocoded in all four levels of degradation. As each of the 200 sentences was only presented once, four experimental groups were formed to counterbalance clarity levels across participants and predictability conditions. Within each experimental group, stimulus presentation was fully randomized.

### Task

In each trial, participants listened to a sentence beginning over headphones while focusing on a central fixation cross on the monitor (see Supplements for experimental setup). The degraded sentence-final word was presented after a delay period of 100 ms. Participants were asked to rate the clarity of the sentence-final word by adjusting a slider bar between 0% (completely unclear) and 100% (completely clear). Participants were then instructed to say the word they understood, cued by a microphone icon in the center of the monitor. After responding, participants were asked to rate the confidence in their response by adjusting a slider bar between 0% (completely unsure) and 100% (completely sure), after which the next trial began. In total, participants completed 200 trials in four experimental blocks of 50 trials each. Before the start of the experiment, participants completed seven practice trials.

### Behavioural analysis

To replicate previous results of task manipulations from a larger sample [35], we performed two-factor repeated-measures ANOVAS on clarity and confidence ratings to examine the effects of predictability levels (high, medium, low, high mismatch) and sensory evidence (channel degradation: 1, 3, 6, 12). Additionally, we performed repeated measures ANOVAs with predictability level as a factor to investigate the impact of sentence predictability on the distribution of behavioral response types (correct responses, no responses, task-based hallucinations). For all ANOVAs, Mauchly’s test was applied to assess sphericity; if violated, degrees of freedom were adjusted using Greenhouse-Geisser estimates. Significant main and interaction effects were further analyzed with Tukey post-hoc tests. Associations with subclinical and cognitive trait markers were investigated using Pearson correlations.

### Computational modelling

We applied a Bayesian Belief updating model to assess the relative weight of high-level semantic prior beliefs (entropy) in comparison to the sensory evidence (noise degradation, surprisal) and how this balance is modulated by individual differences in subclinical (AQ/SPQ) and cognitive markers (VCI). Computational modelling was performed separately for each continuous trait marker. The modelling approach assumes that participants aim to maximize the probability of providing a correct answer. In each trial, this probability takes a prior value after the presentation of the sentence beginning (semantic prior belief) and then undergoes an update to a posterior value after the presentation of the degraded sentence-final word (sensory evidence). This process follows a Bayesian update in a beta-Bernoulli model, where the observation follows a Bernoulli distribution as the participant’s response either matches the presented sentence-final word (coded as 1) or does not (coded as 0). The prior and posterior distributions are modelled as beta distributions, reflecting uncertain probabilities of correctness.

According to Bayes’ rule, the expected correctness probability is updated using a precision-weighted prediction error:

$${P}_{{m}_{i}|{n}_{i}}={P}_{{m}_{i}}+\frac{1}{{\nu }_{{\mbox{i}}}+1}\left({\delta }_{{m}_{i},{n}_{i}}-{P}_{{m}_{i}}\right),$$

where ${P}_{{m}_{i}}$ and ${P}_{{m}_{i}|{n}_{i}}$ represent the prior and posterior cloze probabilities, respectively; ${m}_{i}$ is the participant’s response in trial i, and ${n}_{i}$ is the corresponding sentence-final word. The prior cloze probabilities were estimated based on the norming sample [70], and the posterior cloze probabilities based on the experimental sample. The Kronecker delta ${\delta }_{{m}_{i},{n}_{i}}$ indicates whether ${m}_{i}=\,{n}_{i}$ while $\nu {\mbox{i}} > 0$ denotes the implied number of previous Bernoulli observations [73], which can be directly interpreted as the weight of the prior relative to sensory evidence. The prediction in this model corresponds to the cloze probability of the participant’s response, which is the only available information to the participant before hearing the sentence-final word. The prediction error, which corresponds to the difference of the correctness of the answer (1 or 0) minus the prediction, updates the expected correctness probability by adding it to the prediction after weighting by a factor of 1/(1+ν). ν formalizes the weight of the prior relative to the sensory evidence and is an implied number of previous observations [73]. ${\nu }_{i}$ is modelled on a trial-by-trial basis within a hierarchical linear model that entails the task parameters entropy, cloze probability of the stimulus, and channel number. We further stratified all estimated by the participant’s subjective clarity rating to assess the direct effects. As we expected the prior weight to be modulated by participants’ trait markers, we included a participant-level coefficient in the following hierarchical model for $\log {\nu }_{i}$:

$$\log \left({\nu }_{i}\right)={\beta }_{1j\left[i\right]}+{\beta }_{2j\left[i\right]}* {{{channels}}}_{i} \\ +{\beta }_{3j\left[i\right]}* {{{entropy}}}_{i}+{\beta }_{4j\left[i\right]}{\mathtt{* }}{{cloz}}{{\mathtt{e}}}_{i} \\ +{\beta }_{5j\left[i\right]}* {{{clarity}}}_{i}$$

$${\beta }_{{kj}}=\,{\gamma }_{1k}+{\gamma }_{2k}* {{{spq}}}_{j}$$

$${\gamma }_{{lk}}\sim { N}\left(0,0.2\right),$$

where i represents the trial, and j[i] the participant on trial i. ${\beta }_{{kj}}$ are participant-level coefficients, and ${\gamma }_{{lk}}$ are population-level coefficients. ${\nu }_{i}$ was estimated in logarithmic space as it is bounded below at zero in native space whereas in logarithmic space it is unbounded.

Hamiltonian Monte Carlo sampling (four chains each with 2000 iterations including 1000 warm-up draws) with the rstan software package [74] was used to estimate the posterior distribution of all parameters (${\gamma }_{{lk}}$). Model outcomes are presented in γ coefficients which capture the effects of task parameters ([gamma1,k]: channel numbers, entropy, and cloze probability) on the prior weight ${\nu }_{i}$ and population-level effect are presented in ([gamma2,k]) and show how trait markers modulate the effects of the task parameters on the prior weight ${\nu }_{i}$. To assess model quality, we conducted a simulation-based parameter recovery analysis and posterior predictive checks (see Supplements).

### EEG Analysis

All preprocessing and analysis steps were performed in mne Python (1.5.0 [75]; see Supplements). For the N400 ERP-analysis, the preprocessed data was segmented into epochs from −200–1000 ms around the sentence-final word onset for each of the four predictability conditions (high, medium, low, high mismatch). A baseline correction of the 200 ms preceding the onset was applied. All epochs were automatically repaired or rejected based on local peak-to-peak amplitude, using the 0.4.1 autoreject package [76]. On average 12.2 epochs were dropped (*SD* = 15.37) per participant. The N400 was operationalized as the mean amplitude between 300–500 ms after sentence-final word onset. For each participant, the mean N400 amplitudes were obtained for correct responses by averaging epochs for each of the 15 centro-parietal electrodes (i.e., C3, C1, Cz, C2, C4, CP3, CP1, CPz, CP2, CP4, P3, P1, Pz, P2, P4) for each predictability condition, collapsing across degradation levels. Both the time frame and the selection of electrodes was based on previous literature using related paradigms [77–79].

To investigate alterations in the mean N400 amplitude depending on the trait scores and the four entropy conditions, we used linear mixed-effect models (LMMs). The sentence’s predictability level, the scaled continuous subclinical (SPQ/AQ) and cognitive (VCI) trait markers, and the interaction between predictability condition and the scaled trait markers were included as fixed effects. Treatment coding was applied with the high predictability condition as the baseline. The participant and the electrode position were defined as random intercepts as we did not define hypotheses about spatial distribution or lateralization of effects. The LMMs were carried out separately for AQ, SPQ, and VCI. Tukey adjusted post-hoc tests and simple slope analyses were conducted to follow up main effects and interaction effects.

$${{{\rm{Mean\; N}}}}400\,{{{{\rm{Amplitude}}}}}_{\left[{{{\rm{AQ}}}}/{{{\rm{SPQ}}}}/{{{\rm{VCI}}}}\right]} \\ \sim {{{{\rm{predictability\; level}}}}}_{\left({{{\rm{high}}}},{{{\rm{medium}}}},{{{\rm{low}}}},{{{\rm{high\; mismatch}}}}\right)} \\ * [{{{\rm{AQ}}}}/{{{\rm{SPQ}}}}/{{{\rm{VCI}}}}]+\left(1{{{\rm{|participant}}}}\right)+\left(1{{{\rm{|electrode\; position}}}}\right)$$

To assess whether condition- or trait-related effects extended beyond the N400 window, we analyzed a later time window (600–900 ms post final-word onset) using the same electrode cluster and statistical approach. Details on the R packages used for the analyses can be found in the Supplementary Materials.

## Results

### Behavioral analysis

To replicate the effect of task manipulations reported from a larger sample, we used repeated-measures ANOVAs. Subjective clarity and confidence ratings significantly decreased with increased degradation and lower predictability (clarity: F(5.38,290.78) = 52.37, p < 0.001, ${\eta }_{G}^{2}$ = 0.124; confidence: F(5.66,265.83) = 42.30, p < 0.001, ${\eta }_{G}^{2}$ = 0.143). Words with fewer channels and lower predictability were perceived as less clear, with lower confidence (Tables S4, S6). These findings indicate that the subjective clarity rating not only reflects the sensory precision of the sentence-final word but is also an indirect measure of the precision of the semantic prior belief, as words following precise priors (higher predictability) were rated as clearer regardless of degradation level. Pearson correlation analyses indicated that increasing schizotypy was associated with increased clarity ratings whereas increasing verbal comprehension skills were associated with decreased clarity ratings, indirectly reflecting increased and decreased precision of the precision of semantic prior beliefs (Figure S6). Although these correlations did not reach significance, a similar effect size between schizotypy and clarity ratings was observed in a larger independent sample, suggesting that larger samples may be required to reliably detect these associations.

Task-based hallucinations increased with lower predictability manipulations (F(1.89,102.05) = 199.22, p < 0.001, ${\eta }_{G}^{2}$ = 0.617). Correct responses decreased (F(2.39,129.24) = 553.00, p < 0.001, ${\eta }_{G}^{2}$ = 0.852) and non-responses increased (F(2.25,121.29) = 64.98, p < 0.001, ${\eta }_{G}^{2}$ = 0.199) (Figure S7, Tables S7, S8, S9). This suggests increasing uncertainty, driven by semantic prior manipulations, contributes to task-based hallucinations. No significant correlations were observed between subclinical trait measures and behavioural performance across predictability conditions. However, verbal comprehension ability showed trend-level associations with performance, characterized by more correct responses in high (p = 0.086) and medium (p = 0.071) predictability conditions and fewer correct responses in low predictability conditions (p = 0.071), suggesting a potential interaction between verbal ability and contextual uncertainty.

### Computational modelling

We performed a Bayesian belief updating model separately for each trait marker on the behavioral task data to investigate a potential imbalance in the weighting of high-level semantic prior beliefs and sensory evidence. A linear model including the trial-based task parameters (i.e., the sentence beginning’s entropy, sentence-final word’s cloze probability, the channel number with which the sentence-final word was presented, and the subjective clarity rating) was used to estimate the strength ν of the prior weight depending on the relative trait marker.

**Fig. 3:**
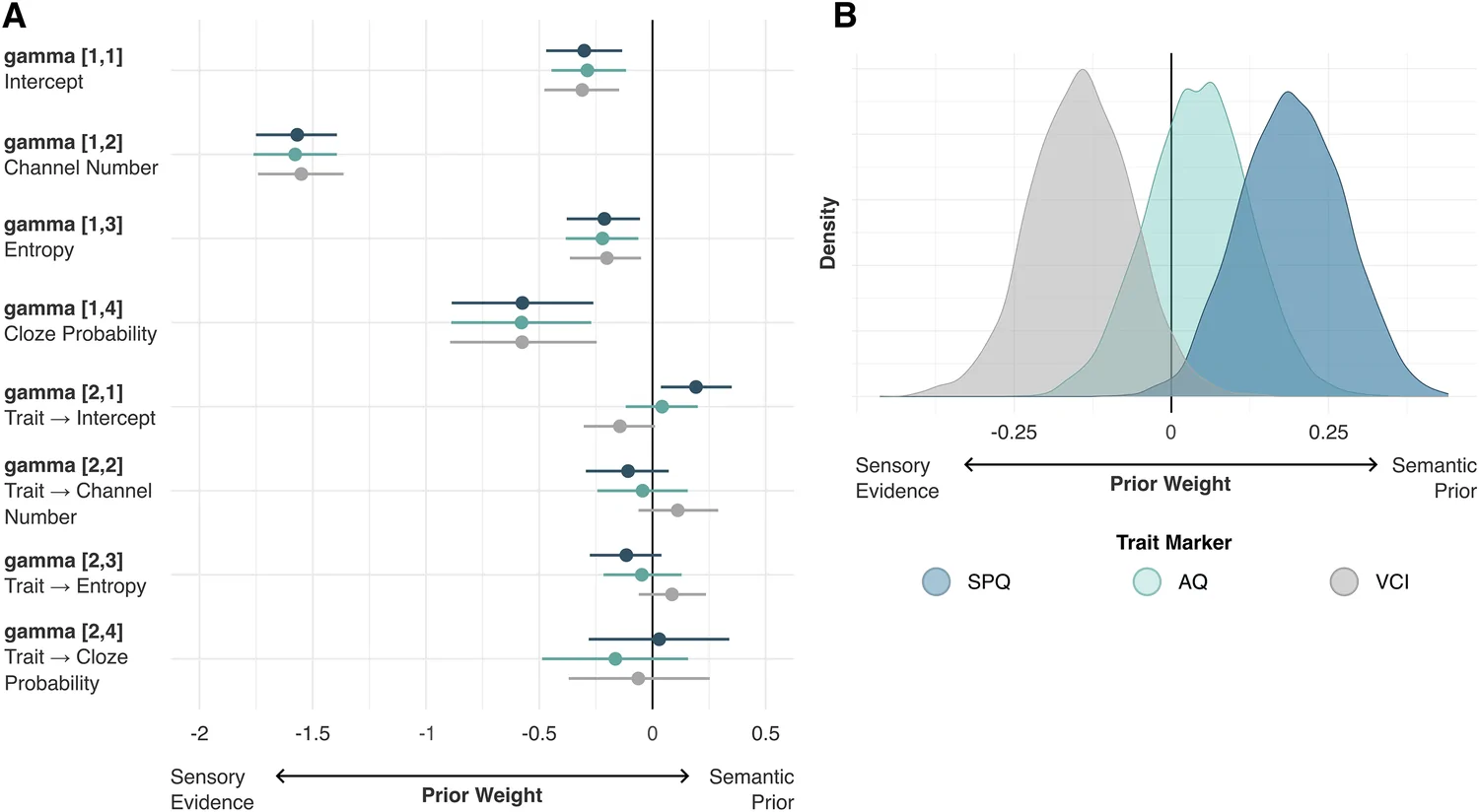
Computational Modelling Results. **A** For each trial, a Bayesian belief updating model was applied to estimate the weight ν of the semantic prior beliefs relative to sensory evidence. Trial-level values of ν were then explained using a hierarchical linear model with task parameters (channel number, entropy, cloze probability) as predictors whose effects were further modulated by the participants’ individual trait scores. Parameters with 95% intervals excluding 0 indicate a significant effect. For example, gamma[1,2], captures the effect of channel degradation on ν, reflecting stronger weighting of sensory evidence with increasing channel number (i.e., clearer acoustic input). Gamma[2,2] quantifies trait-related modulation of ν using the population-level predictors (SPQ, AQ, VCI). Here, increasing SPQ was associated with a significant shift towards the semantic prior, indicating prior overweighting. **B** Differences in the distributions of the effect of SPQ, AQ, and VCI on the prior weight. SPQ Schizotypal Personality Questionnaire [62, 63], AQ Autism Spectrum Quotient [64], VCI Verbal Comprehension Index derived from the WAIS-IV [65].

The model estimation converged and met all quality checks (Figure S11 and S12). Table 1 and Fig. 3 show the parameter estimates using SPQ, AQ and VCI as model predictors. The modelling results showed that increasing SPQ induced a significant shift in the weighting to the prior belief (coefficient gamma[2,1]), while AQ and VCI did not significantly alter the prior weight. Specifically, increasing SPQ by 10 points (range 0–296) induced an increase of 3.8% in the weighting of semantic prior beliefs relative to the sensory evidence (i.e., exp(−0.30 + 0.19/50.13)/exp(−0.30) = 1.0038, where 50.13 is SPQ SD, −0.30 is the gamma[1,1] intercept, and 0.19 is gamma[2,1], reflecting SPQ impact). Distribution analyses of the trait marker parameter gamma[2,1] using Tukey corrected Games-Howell post-hoc tests indicated that the effect of the trait markers on the prior weight was significantly different between all trait markers. The effect of SPQ was significantly stronger than the effect of AQ (0.15, 95% CI [0.15, 0.15], p < 0.001). The effect of VCI was significantly weaker than the effect of SPQ (−0.34, 95% CI [−0.34, −0.33], p < 0.001) and AQ (−0.19, 95% CI [−0.19, −0.18], p < 0.001). Parameter recovery analyses revealed good parameter recovery for all trait-level parameters and posterior predictive checks indicated good agreement between the model-implied predictions (see Supplements).

**Table 1:** Parameter Estimates of Gammas.

Parameter	Trait	Median	MAD	CI 2.5%	CI 25%	CI 50%	CI 75%	CI 97.5%	n_eff	Rhat
**gamma[1,1]** Intercept	SPQ	−0.30	0.08	−0.46	−0.36	−0.30	−0.24	−0.14	6312.26	1.00
	AQ	−0.29	0.08	−0.45	−0.34	−0.29	−0.23	−0.12	6156.85	1.00
	VCI	−0.31	0.08	−0.48	−0.37	−0.31	−0.26	−0.15	6459.19	1.00
**gamma[1,2]** Channel Number	SPQ	−1.57	0.10	−1.76	−1.64	−1.57	−1.51	−1.38	6679.80	1.00
	AQ	−1.58	0.09	−1.76	−1.64	−1.58	−1.52	−1.39	7927.03	1.00
	VCI	−1.55	0.10	−1.74	−1.62	−1.55	−1.48	−1.36	6856.78	1.00
**gamma[1,3]** Entropy	SPQ	−0.21	0.08	−0.37	−0.26	−0.21	−0.15	−0.05	6753.26	1.00
	AQ	−0.22	0.08	−0.38	−0.28	−0.22	−0.17	−0.06	7356.98	1.00
	VCI	−0.20	0.08	−0.36	−0.26	−0.20	−0.15	−0.05	7709.20	1.00
**gamma[1,4]** Cloze Probability	SPQ	−0.58	0.16	−0.89	−0.68	−0.58	−0.47	−0.26	6996.01	1.00
	AQ	−0.58	0.16	−0.89	−0.69	−0.58	−0.47	−0.27	7042.08	1.00
	VCI	−0.58	0.17	−0.89	−0.69	−0.58	−0.46	−0.25	7811.48	1.00
**gamma[2,1]** Trait Intercept	SPQ	0.19	0.08	0.04	0.14	0.19	0.25	0.36	6619.53	1.00
	AQ	0.04	0.08	−0.12	−0.01	0.04	0.10	0.20	6366.04	1.00
	VCI	−0.14	0.08	−0.30	−0.20	−0.14	−0.09	0.01	6409.05	1.00
**gamma[2,2]** Trait → Channel Number	SPQ	−0.11	0.09	−0.29	−0.17	−0.11	−0.05	0.07	7402.99	1.00
	AQ	−0.04	0.10	−0.24	−0.11	−0.04	0.02	0.16	8020.09	1.00
	VCI	0.11	0.09	−0.06	0.05	0.11	0.17	0.29	6828.73	1.00
**gamma[2,3]** Trait → Entropy	SPQ	−0.12	0.08	−0.27	−0.17	−0.12	−0.06	0.04	7055.87	1.00
	AQ	−0.05	0.09	−0.22	−0.11	−0.05	0.01	0.13	8404.71	1.00
	VCI	0.09	0.07	−0.06	0.04	0.09	0.13	0.24	7619.20	1.00
**gamma[2,4]** Trait → Cloze Probability	SPQ	0.04	0.16	−0.28	−0.07	0.04	0.14	0.34	6642.48	1.00
	AQ	−0.16	0.16	−0.49	−0.27	−0.16	−0.05	0.16	7650.75	1.00
	VCI	−0.06	0.16	−0.37	−0.17	−0.06	0.04	0.25	6402.44	1.00

The prior weight was stratified by the effect of the clarity rating. As the posterior gamma distribution of clarity is uninterpretable, it is not presented in the table.

*MAD* Median absolute deviation, *n_eff* effective sample size, of 10000 samples have been drawn, *Rhat* potential scale reduction factor on split chains; Rhat<1.04 indicates convergence, *SPQ* Schizotypal Personality Questionnaire [62, 63], *AQ* Autism Spectrum Quotient [64], *VCI* Verbal Comprehension Index derived from the WAIS-IV [65].

### N400 analysis

**Fig. 4:**
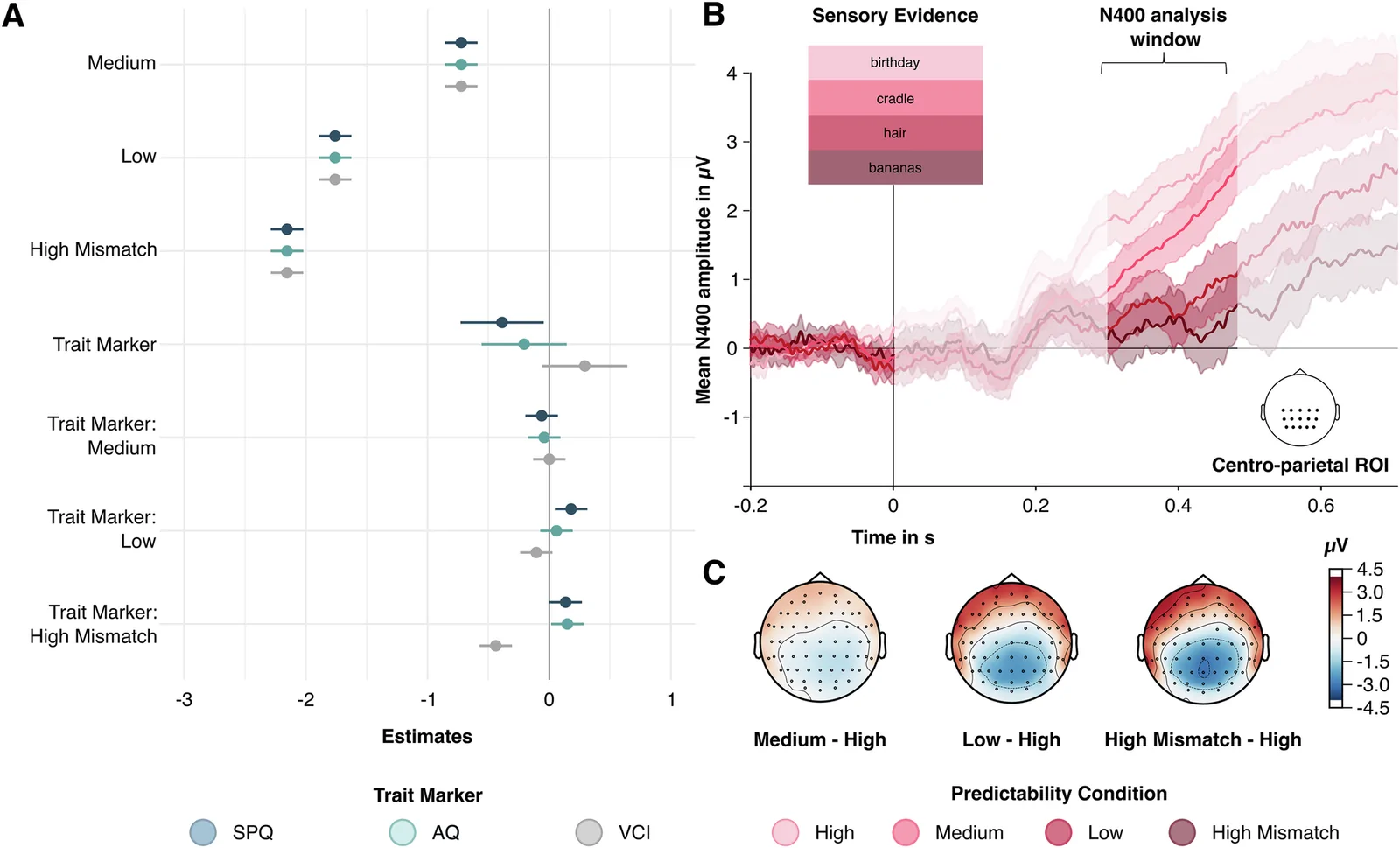
N400 Analysis Results. **A** Linear mixed model results. Impact of subclinical (SPQ/AQ) and cognitive (VCI) trait markers and sentence entropy level on mean N400 amplitude. **B** ERP Visualization. Mean N400 amplitude for sentence entropy levels time-locked to the onset of the sentence final word with a 200 ms pre-stimulus baseline period (e.g., high predictability: She wished her a happy birthday, medium predictability: The baby slept in the cradle, low predictability: In the bathtub swam a hair, high mismatch predictability: He decorated the room with bananas; **C** Topographical Scalp Maps. Topography of the mean amplitude 300–500 ms of the difference wave for the medium vs. high predictability (left), the low vs. high predictability condition (middle) and the high mismatch vs. high predictability condition (right); SPQ: Schizotypal Personality Questionnaire [62, 63]; AQ: Autism Spectrum Quotient [64]; VCI: Verbal Comprehension Index derived from the WAIS-IV [65].

We performed LMMs separately for the subclinical and cognitive trait markers to investigate the effect of the trait markers and sentence predictability levels on the mean N400 amplitude. Sentence predictability levels (high, medium, low, high mismatch), subclinical (SPQ/AQ) and cognitive (VCI) trait markers, and their interactions were included as fixed effects, while the individual participant and 15 centro-parietal electrode positions were included as random intercepts. The results are depicted in Table 2 and Fig. 4.

**Table 2:** N400 LMM Results.

	SPQ			AQ			VCI		
*Predictors*	*Estimates*	*CI (95%)*	*P-Value*	*Estimates*	*CI (95%)*	*P-Value*	*Estimates*	*CI (95%)*	*P-Value*
Intercept (Baseline: High)	2.48	1.71 – 3.26	**<0.001**	2.48	1.70 – 3.27	**<0.001**	2.48	1.70 – 3.27	**<0.001**
Medium	−0.72	−0.86 – −0.59	**<0.001**	−0.72	−0.86 – −0.59	**<0.001**	−0.72	−0.85 – −0.59	**<0.001**
Low	−1.76	−1.89 – −1.63	**<0.001**	−1.76	−1.89 – −1.63	**<0.001**	−1.76	−1.89 – −1.63	**<0.001**
High Mismatch	−2.16	−2.29 – −2.02	**<0.001**	−2.16	−2.29 – v2.02	**<0.001**	−2.16	−2.29 – −2.02	**<0.001**
Trait (Baseline: High)	−0.40	−0.74 – −0.05	**0.027**	−0.21	−0.56 – 0.14	0.250	0.29	−0.06 – 0.64	0.100
Trait: Medium	−0.06	−0.20 – 0.07	0.403	−0.04	−0.17 – 0.09	0.553	0.00	−0.13 – 0.13	0.985
Trait: Low	0.18	0.05 – 0.31	**0.008**	0.06	v0.07 – 0.19	0.376	−0.11	−0.24 – 0.03	0.120
Trait: High Mismatch	0.15	0.02 – 0.28	**0.028**	0.15	0.02 – 0.28	**0.028**	−0.44	−0.57 – −0.31	**<0.001**
**Random Effects**									
σ^2^	1.92			1.93			1.90		
τ_00_	1.54 _participant_			1.62 _participant_			1.62 _participant_		
	1.91 _electrodeposition_			1.91 _electrodeposition_			1.91 _electrodeposition_		
ICC	0.64			0.65			0.65		
N	15 _electrodeposition_			15 _electrodeposition_			15 _electrodeposition_		
	55 _participant_			55 _participant_			55 _participant_		
Observations	3300			3300			3300		
Marginal R^2^ / Conditional R^2^	0.135 / 0.691			0.121 / 0.690			0.125 / 0.695		

All main and interaction effects are depicted in reference to the high predictability condition. For follow-up analyses, Tukey post-hoc tests and simple slope analyses were conducted.

For all three LMMs, there was a significant main effect of sentence predictability level in reference to the high predictability baseline condition. Paired Tukey corrected post-hoc comparison revealed a graded effect of predictability with a more negative N400 amplitude in the high mismatch compared to the low predictability condition (SPQ/AQ/VCI: −0.40, 95% CI = [−0.57, −0.22]); a more negative N400 amplitude in the low compared to the medium predictability condition (SPQ/AQ/VCI: −1.04, 95% CI = [−1.21, −0.86]), and a more negative N400 amplitude in the medium compared to the high predictability condition (SPQ/AQ/VCI:−0.72, 95% CI = [−0.90,−0.55]).

With increasing SPQ, there was a significant negative effect on the mean N400 amplitude in the high predictability baseline condition, indicating that the mean N400 amplitude is decreasing with increasing SPQ. Additionally, we found a significant interaction effect between SPQ and the low and high mismatch predictability condition compared to the effect of SPQ in the baseline condition, indicating that the mean N400 difference between the high mismatch and the high predictability condition and the mean N400 amplitude difference between the low and the high predictability condition decreased with increasing SPQ (Table 2). Follow-up simple slope analyses confirmed that there was a significant decrease in mean N400 amplitude with increasing SPQ in the high predictability condition (−0.40, SE = 0.17, t = −2.28, *p* = 0.027). In addition, the mean N400 amplitude also significantly decreased in the medium predictability condition (−0.45, SE = 0.17, t = −2.60, *p* = 0.012). There was no significant change in the low (−0.22, SE = 0.17, t = −1.24, *p* = 0.221) and high mismatch (−0.25, SE = 0.17, t = −1.42, *p* = 0.162) predictability condition with increasing SPQ on their own.

For the LMM including AQ, there was a significant interaction effect between AQ and the high mismatch condition compared to the effect of AQ in the baseline condition, suggesting that the mean N400 difference between the high and high mismatch condition decreased with increasing AQ. Follow-up simple slope analyses did not reveal a significant change of the mean N400 amplitude with increasing AQ in any condition on their own (high: −0.21, SE = 0.18, t = −1.15, *p* = 0.254; medium: −0.25, SE = 0.18, t = −1.38, *p* = 0.173; low: −0.15, SE = 0.18, t = −0.81, *p* = 0.420; high mismatch: −0.06, SE = 0.18, t = −0.31, *p* = 0.757).

For the LMM including VCI, there was a significant interaction effect between VCI and the high mismatch condition compared to the effect of VCI in the baseline condition, indicating an increasing mean N400 difference between the high and high mismatch predictability condition with increasing VCI. Follow-up simple slope analyses did not reveal a significant change of the mean N400 amplitude with increasing VCI in any condition on their own (high: 0.29, SE = 0.18, t = 1.64, *p* = 0.106; medium: 0.295, SE = 0.18, t = 1.65, *p* = 0.104; low: 0.188, SE = 0.18, t = 1.05, *p* = 0.297; high mismatch: −0.145, SE = 0.18, t = v0.82, *p* = 0.419).

For all LMMs, post-hoc simulation-based power analyses were conducted to evaluate the detectability of the interaction between predictability condition and clinical trait. The estimated power to detect this interaction at α = 0.05 was 93.70% (95% CI [93.70, 95.13]) for SPQ, 70.40% (95%CI [67.46, 73.22]) for AQ, and 100% (95% CI [99.63, 100]) for VCI.

LMM analyses of a later time window (600–900 ms) indicated that the modulation observed during the N400 window extended into subsequent processing stages, with high-predictability sentences eliciting more positive mean amplitudes than both low-predictability and high-predictability mismatch sentences. Comparable modulations by trait markers were also observed in this later time frame (see Supplementary Materials).

## Discussion

This study aimed to improve the computational and neurophysiological understanding of language processing deficits in subclinical expressions of ASD and SSD within the framework of predictive processing. Using an auditory comprehension task, we manipulated the precision of semantic prior beliefs and sensory evidence and applied computational modeling and EEG, focusing on semantic prediction errors reflected in the N400 component. Computational modeling revealed that increasing schizotypal traits were associated with a significant overweighting of prior beliefs, while autistic traits did not show a significant shift. The schizotypy-related overweighting of semantic prior beliefs was reflected in a reduced semantic prediction error signal, indexed by smaller N400 differences between highly predictable and both highly unpredictable and highly predictable sentences which were completed by an unexpected sentence-final word. A similar pattern emerged for increasing autistic traits, though the effect was less distinct as only the N400 difference between highly predictable and highly predictable mismatch sentences decreased.

Consistent with the ASD-SSD predictive processing continuum hypothesis, we found that increasing schizotypy was related to an overweighting of high-level semantic prior beliefs. This aligns with both computational [33–38] and sine-wave speech studies [80–82], which found stronger prior weighting and overly strong priors for perceiving speech in SSDs. Electrophysiologically, this shift manifested in a reduced prediction error signal, reflected in an attenuated mean N400 amplitude difference between high predictability and both high mismatch and low predictability sentences. This aligns with previous findings from semantic priming paradigms indicating reduced N400 effect across illness stages [56–58]. By using an auditory sentence processing design, our findings add to the limited number of studies which have moved beyond word-pair designs [77, 79, 83], demonstrating that impaired use of semantic context also affects higher-level auditory processing. Notably, we found that the reduced N400 effect with increasing schizotypy was driven specifically by impaired processing of predictable input as N400 amplitudes became more negative for sentence-final words which matched which high and medium predictable sentence contexts. This aligns with previous semantic priming literature which identified more negative amplitudes for related compared to unrelated word pairs as the source of a diminished N400 effects [79, 84–86]. One possible explanation for the altered processing of predictable input is that individuals with increasing schizotypal traits assign greater precision to semantic prior beliefs which are less accurate. Consequently, when the presented sentence-final word deviates from a highly precise but inaccurate prediction, a larger N400 amplitude would be expected, particularly in highly predictable sentence contexts where semantic predictions are most precise. By introducing multiple levels of contextual predictability, our results show that these alterations are not restricted to highly predictable associations but also occur in sentences that provide only moderate predictive cues. Relatedly, we have shown that individuals with higher schizotypal and also autistic traits form less stable semantic predictions, which also specifically affects highly predictable sentence contexts [87].

In contrast to the proposed ASD-SSD predictive processing continuum [9–11], we did not observe sensory overweighting with increasing autistic traits. Evidence for such overweighting in ASD has largely emerged from perceptual [13–15, 20, 21, 23] and social decision-making tasks [16, 17, 22, 24] and remains overall heterogeneous [88]. Therefore, it may be possible that predictive processing impairments in ASD are domain-specific, with language processing remaining intact. However, not only robust impairments in speech processing in ASD [40] but also our electrophysiological findings argue against this explanation as we observed attenuated prediction error signaling as indexed by smaller mean N400 amplitude difference between highly predictable sentences which were completed by predictable versus unpredictable sentence-final words. In comparison to schizotypy, however, post-hoc analysis could not identify the driving condition of this effect. This result is consistent with previous studies reporting reduced rather than enhanced N400 effects in ASD [52–55] which would be expected for sensory overweighting even though the evidence is more heterogeneous than for SSD [47–49, 51]. Although computational modeling did not reveal altered weighting, the neurophysiological results suggest a subtle overweighting of prior beliefs that may only emerge under specific task conditions, similar but weaker than the pattern seen with increasing schizotypy.

Overall, our findings do not provide evidence for diametrically opposed predictive processing profiles in subclinical expressions of ASD and SSD. Instead, both trait dimensions were associated with attenuated N400 prediction error responses, suggesting a shared tendency to overweight semantic prior beliefs. Together with our previous findings [87], these results indicate that both subclinical spectra show comparable difficulties in flexibly using contextual information to guide language comprehension. This interpretation aligns with psychometric evidence indicating that ASD and SSD share substantial overlap in symptom domains [1, 89–91] which is mirrored in the relationship between autistic and schizotypal personality traits [4, 5] and replicated in our subclinical sample (R = 0.51). Psychotic symptoms (i.e., hallucinations and delusions) may more clearly differentiate ASD and SSD which could explain why perceptual paradigms often identify diametrical predictive processing alterations. However, other studies demonstrate significant psychotic vulnerability in ASD [3, 92], suggesting that the relationship between ASD and SSD is more complex than a single predictive processing continuum can capture. Empirical work in SSD further indicates that multiple predictive processing alterations can coexist, including low-level sensory overweighting alongside overweighting of high-level priors. These mechanisms may map onto different symptoms and rely on distinct neurobiological substrates [7, 8]. A similarly domain-specific framework may help explain the heterogeneous evidence for sensory overweighting in ASD and provide a more nuanced account of its computational phenotype.

Finally, we also examined whether verbal comprehension abilities influence the balance between prior beliefs and sensory evidence. Although computational modeling did not reveal a significant shift, higher verbal abilities showed a trend toward greater reliance on sensory information. Electrophysiologically, this was reflected in an enhanced difference between the processing of predictable versus unpredictable sentence-final words following highly predictable sentences, indicating a larger prediction error response. This aligns with previous evidence, linking larger N400 effects to individual differences in statistical learning [93], working-memory performance [94], and language skills [95]. These findings suggest that verbal abilities may help shaping a most efficient predictive framework with reliable semantic predictions, enabling greater sensitivity to unexpected input. Importantly, there was no association between schizotypy and verbal comprehension skills, indicating that prior overweighting in schizotypy cannot be explained by reduced verbal ability.

### Limitations, control analyses and future directions

We focused on the N400 component, given its link to semantic prediction error processing [45, 46]. Visual inspection additionally suggested effects at later latencies, potentially indicative of modulations in the P600 or other late positive components. Although the P600 has been classically associated with syntactic processing, accumulating evidence shows that it is also sensitive to semantic processing and higher-level semantic integration processes [59–61]. Alterations in late positive components have also been identified alterations in SSD und subclinical cohorts [79, 96, 97]. In the present sample, however, control analyses indicated that the modulation observed during the N400 window extended into later time periods, with high predictability sentences eliciting more positive mean amplitudes than low and high predictability mismatch sentences. This pattern is inconsistent with previous P600 findings, which would predict increased positivity for semantic unpredictability or violations. One possibility is that the robust N400 effect overlapped with or masked later positive components. Alternatively, the absence of a distinct P600 may relate to paradigm differences as previous studies often used designs with multi-sentence context which may require stronger semantic integration [59–61, 79]. More fine-grained analyses of sentence types, time windows, and spatial distributions may help disentangle early semantic prediction error effects from later integrative processes, but this will require further paradigm optimization.

In addition, we observed trend-level associations between task performance and subclinical trait measures, suggesting a potential relationship between behavior and individual trait differences. However, larger samples will be required to investigate the relationship in more detail.

A strength of the experimental paradigm was the independent manipulation of the precision of semantic prior beliefs through entropy and sensory precision through auditory degradation and contextual surprisal which allowed to computationally assess the relative weighting of prior beliefs and sensory evidence. For the ERP analysis, trials were averaged by sentence predictability for correct responses, collapsing across auditory degradation levels as trial numbers for correct responses per degradation level were too low to allow for a reliable separate analysis. Previous research suggests that auditory clarity may modulate the latency and the magnitude of the N400 component in addition to predictability effects which are considered the primary driver of these effects [98, 99]. As our computational modelling approach also includes this manipulation, future analyses combining single-trial ERP analyses with computational parameters may allow a more fine-grained investigation of how sensory and prior precision jointly shape neural responses. Importantly, degradation levels were counterbalanced across sentences and participants, reducing the likelihood that the effects were driven by systematic sensory differences.

Finally, as the present study was based on a healthy subclinical sample of 55 individuals in which schizotypal and autistic traits were strongly correlated, the ability to dissociate disorder-specific mechanisms may have been limited. Although the strength of this relationship is consistent with previous findings from larger-scale studies [4, 5], future research using larger samples and clinical cohorts will be needed to better disentangle disorder-specific mechanisms and account for the heterogeneity of ASD and SSD. Whole-brain modeling and advanced single-trial approaches may further help to directly map computational parameters onto electrophysiological signatures.

## Supplementary information

Supplements

## Data Availability

Data and code may be accessed through: https://osf.io/3rv4m/overview.
