# Supplementary material for: Altered semantic prediction error processing with increasing schizotypal and autistic traits
Source: Transl Psychiatry. 2026 Sep 30;16:502. doi: 10.1038/s41398-026-04479-4 (PMC13627722; doi:10.1038/s41398-026-04479-4)
Supplement: Supplementary file 1 [file 41398_2026_4479_MOESM1_ESM.docx]

**Altered Semantic Prediction Error Processing with Increasing Schizotypal and Autistic Traits**

**Supplements**

Elisabeth F. Sterner (M.Sc.)^1,2,3*^, Verena F. Demler^1,2^, Rachel Lenz (PhD)^4^, Lucy J. MacGregor (PhD)^5^, Christoph Mathys (PhD)^6^, Franziska Knolle (PhD)^1,2*^

1 School of Medicine and Health, Department of Diagnostic and Interventional Neuroradiology, Technical University of Munich, Munich, Germany

2 School of Medicine and Health, TUM-NIC Neuroimaging Center, Technical University of Munich, Munich, Germany

3 Department of Experimental Psychology, Ludwig-Maximilians University Munich, Munich, Germany

4 School of Medicine and Health, Department of Anesthesiology and Intensive Care, Technical University of Munich, Munich, Germany

5 MRC Cognition and Brain Sciences Unit, University of Cambridge, Cambridge, UK

6 Interacting Minds Centre, Aarhus University, Denmark

*corresponding author:

Elisabeth Sterner/Franziska Knolle

Department of Diagnostic and Interventional Neuroradiology, School of Medicine and Health, Klinikum rechts der Isar, Technical University Munich, 81675 Munich, Germany

Phone: +49 89 4041 7966	Fax: +49 89 4140 4653

Email: elisabeth.sterner@tum.de/franziska.knolle@tum.de

ORCID: 0009-0009-9638-2624/0000-0002-9542-613X

**Supplementary Methods**

**Experimental Setup**

During the experiment, participants were seated centrally in front of a monitor (BenQ Corporation 24’’, resolution 1920x1080) in a darkened room and wore headphones (hama®, sensitivity 105 ± 3dB, frequency range 20Hz–20kHz, impedance 32W) with an integrated microphone (hama®, sensitivity -42 ±3dB, frequency range 50Hz–10kHz, impedance 2200 W, directivity omnidirectional) for stimulus presentation and recording. The experimental task was created using Psychopy3 Experiment Builder (v2022.1.1;^1^) and presented in Psychopy Coder (v2022.1.1) which was synchronized with the recording of the EEG signals in BrainVision Recorder 1.24.0001 (Brain Products GmBH, Gilching, Germany).

**Task specifics**

| **Table S1:** Properties of task material | | | |
| --- | --- | --- | --- |
| **Entropy Condition** | **Entropy** | **Cloze Probability** | **Sentence Length** |
|  | **Mean**  **(SD)** | **Mean**  **(SD)** | **Mean**  **(SD)** |
| Low | 0.28  (0.21) | 0.93  (0.06) | 6.72  (1.26) |
| Medium | 1.47  (0.29) | 0.54  (0.07) | 6.71  (1.78) |
| High | 2.53  (0.35) | 0.05  (0.01) | 6.95  (1.36) |
| Low Mismatch | 0.39  (0.25) | 0.00  (0.00) | 7.05  (2.12) |

**Computational Modelling**

Parameter Recovery Analysis

To assess the identifiability of the model parameters, we conducted a simulation-based parameter recovery analysis separately for each trait marker. For each simulation, we generated 50 synthetic datasets using the empirical predictor structure of the original data, including channels, entropy, cloze probability, clarity, and the respective trait measures. Generating values for the population-level model parameters were sampled from a normal distribution, (N(0, 0.15)), and used to compute trial-wise response probabilities according to the model equations. Binary response outcomes, indicating whether the simulated response matched the sentence-final target word, were then sampled from a Bernoulli distribution. Each simulated dataset was refitted using the same model specification and estimation procedure as in the main analysis. Specifically, models were estimated using Hamiltonian Monte Carlo sampling with 4 chains, 2000 iterations per chain, 1000 warmup iterations, and an adapt_delta of .90. Recovered posterior mean estimates of the population-level parameters were compared with the corresponding generating values using Pearson correlation, mean bias, root mean squared error (RMSE), and the proportion of generating values falling within the 95% posterior credible interval. The parameter recovery analysis indicated good recoverability of the model parameters, with a high correlation between generating and recovered parameter values, low mean bias, and close-to-nominal 95% credible-interval coverage.

**Posterior Predictive Checks**

To evaluate whether the fitted model could reproduce the observed behavioral patterns, posterior predictive checks were performed. Using the posterior samples from the fitted models, trial-wise model-implied probabilities of correct responses were extracted and averaged across posterior draws. These predicted probabilities were then compared with the observed proportion of correct responses at the participant level and across entropy, clarity, cloze probability, and the trait measures. This allowed us to investigate whether the model captured both individual differences in overall task performance and the central behavioral patterns observed across experimental conditions and trait variation. As an additional assessment of model fit, we examined the trial-wise log-likelihood of the observed responses under the fitted model. This quantified how much probability the model assigned to the responses that were actually observed, with higher log-likelihood values indicating better predictive fit.

**EEG Recording and Preprocessing**

EEG was continuously recorded using a MR-compatible EEG 10-10 system with 64 Ag/AgCl ring electrodes (BrainVision Recorder, version 1.24.0001, Brain Products GmbH, Gilching, Germany) with a 1000Hz sampling rate and an online low pass filter of 250Hz. FCz was used as an active reference (online) and AFz as the ground. Impedance values were kept below 10kΩ throughout the experiment. EEG signals were amplified using a BrainAmp MRplus amplifier (BrainAmp, Brain Products GmbH, Gilching, Germany) in the range of ±3.28mV at a resolution of 0.1μV.

During preprocessing, the ECG electrode was dropped for all participants due to noise. A zero-phase FIR filter was applied (band pass: 0.1-40Hz, lower and upper transition bandwidth: 0.5Hz). A notch filter was applied at 50Hz and the harmonics 100Hz and 150Hz. Automatic break detection was applied with a minimum break detection of 20s with a minimum gap of 2s after the previous break and before the next break. The EEG data was then rereferenced using a common average reference. To remove ocular artifacts, independent component analysis (ICA) was performed using the Picard algorithm (maximum 1000 iterations). The data were band-pass filtered between 1 and 30 Hz before ICA fitting. Eye-movement-related components were identified by correlating independent components with signals from Fp1 and F8, serving as virtual electrooculogram channels for vertical and horizontal eye movements, respectively. Identified components were removed prior to further analysis.

**Statistical Analysis**

Statistical analyses were performed in R Studio^2^ and visualized using the ggplot2 package, version 3.5.1^3^, and the sjPlot package, version 2.8.17^4^. To investigate alterations in the mean N400 amplitude depending on the clinical scores and the four entropy conditions, we used linear mixed-effect models (LMMs) performed with the lme4 package, version 1.1 – 35.5^5^. Tukey adjusted post hoc tests and simple slope analyses were conducted to follow up main effects and interaction effects using the emmeans package, version 1.10.4^6^, and the interactions package, version 1.2.0^7^. Post hoc simulation based power analysis were performed using the simr package, version 1.0.7^8^, with 1000 simulations using likelihood ratio tests comparing the full model to a reduced model without the interaction term of interest.

**Supplementary Results**

**Computational Modelling**

Parameter Recovery

Parameter recovery is summarized in Table S2 and visualized in Figure S1. All parameters show good recoverability with all Pearson correlations between true and recovered parameter values being highly significant (p < .001).

| **Table S2:** **Simulation-based Parameter Recovery Results** | | | | |
| --- | --- | --- | --- | --- |
| **Model** | **Pearson correlation** | **Mean bias** | **RMSE** | **95% CI coverage** |
| SPQ | 0.92 | 0.00 | 0.06 | 0.97 |
| AQ | 0.91 | 0.00 | 0.07 | 0.97 |
| VCI | 0.91 | 0.00 | 0.07 | 0.96 |
| **Notes.** SPQ: Schizotypal Personality Questionnaire^65,66^; AQ: Autism Spectrum Quotient^67^; VCI: Verbal Comprehension Index derived from the WAIS-IV^68^, RMSE: Root mean squared error | | | | |

**Figure S1.** Scatter plots between true and recovered model parameters for computational models including A) SPQ, B) AQ, C) VCI.

R: Pearson Correlation Coefficient; SPQ: Schizotypal Personality Questionnaire^9,10^; AQ: Autism Spectrum Quotient^11^; VCI: Verbal Comprehension Index derived from the WAIS-IV^12^

Posterior Predictive Checks

Posterior predictive checks indicated that the model-implied posterior prediction probabilities captured the observed behavioural responses well (Figure S2). We performed logistic regressions predicting observed trial-wise correctness from the posterior mean predicted probabilities. The resulting pseudo-$R^{2}$ were 0.99 for the model including SPQ, 0.99 for the model including AQ, and 0.99 for the model including VCI, indicating good agreement between model-implied and observed behavioural responses. In addition, the computational models reproduced the observed behavioural patterns across entropy, cloze probability, and trait levels (Figure S3-S5), indicating that the fitted models reproduced the relationship between the trial-level and subject-specific predictors and behavioural performance.

**Figure S2.** Relationship between the model-implied posterior probability of a correct response and the observed trial-wise behavioural performance. SPQ: Schizotypal Personality Questionnaire^9,10^; AQ: Autism Spectrum Quotient^11^; VCI: Verbal Comprehension Index derived from the WAIS-IV^12^

**Figure S3.** Posterior predictive check by quartiles of entropy. Comparison of the observed mean probability of a correct response (solid line) and the model-implied posterior mean probability of a correct response (dashed line). SPQ: Schizotypal Personality Questionnaire^9,10^; AQ: Autism Spectrum Quotient^11^; VCI: Verbal Comprehension Index derived from the WAIS-IV^12^

**Figure S4.** Posterior predictive check by quartiles of cloze probability. Comparison of the observed mean probability of a correct response (solid line) and the model-implied posterior mean probability of a correct response (dashed line). SPQ: Schizotypal Personality Questionnaire^9,10^; AQ: Autism Spectrum Quotient^11^; VCI: Verbal Comprehension Index derived from the WAIS-IV^12^

**Figure S5.** Posterior predictive check by quartiles of trait markers. Comparison of the observed mean probability of a correct response (solid line) and the model-implied posterior mean probability of a correct response (dashed line). SPQ: Schizotypal Personality Questionnaire^9,10^; AQ: Autism Spectrum Quotient^11^; VCI: Verbal Comprehension Index derived from the WAIS-IV^12^

**Statistical results showing the impact sentence entropy and target word degradation on clarity and confidence rating**

Clarity rating

We performed a two-factor repeated measure ANOVA to examine the effect of the entropy channel manipulation on mean subjective confidence ratings. Mauchly’s test indicated that the assumption of sphericity had been violated for the main effects of condition (W = 0.63, p < .001, ε = .65) and channel number (W = 0.67, p < .05, ε = .70) and for the interaction effect of condition and channel number (W = 0.60, p < .001, ε = .67). Consequently, degrees of freedom were corrected using Greenhouse-Geisser estimates of sphericity. All effects reported are significant at p < .05. There was a significant main effect of entropy condition, a main effect of channel number as well as an interaction effect of condition and channel number on the mean clarity rating (see **Table S3**). Tukey post hoc tests of the significant interaction effect revealed that for channel number 12, 6, and 3, the mean clarity rating significantly decreased from low entropy to medium entropy, to high entropy sentences, with the target word of the low entropy mismatch condition being perceived as the most unclear (see **Table S4**). For the 12 channel condition, the target word of a low and medium entropy sentence was perceived as clearer than during the high entropy or low entropy mismatch condition, however there was no significant difference between the low and medium entropy condition. For the 1 channel condition, the target word of a low entropy sentence was perceived as clearer than during the high entropy or low entropy mismatch condition, however there was no significant difference between the medium entropy condition, and between the medium and low entropy mismatch condition.

Pearson correlation analysis between subjective clarity ratings and subclinical and cognitive trait markers are depicted in **Figure S6**.

| **Table S3: Results of the repeated measures ANOVA investigating the effect of condition and channel number on the mean clarity rating** | | | | | |
| --- | --- | --- | --- | --- | --- |
| **Effect** | **df** | **MSE** | **F** | **ges** | **p-value** |
| condition | 1.88, 101.67 | 109.14 | 285.31 *** | .279 | <.001 |
| channel_nr | 2.02, 108.93 | 282.50 | 1134.91 *** | .810 | <.001 |
| condition:channel_nr | 5.38, 290.78 | 75.86 | 52.37 *** | .124 | <.001 |

| **Table S4: Results of Tukey post hoc tests investigating the interaction effect of entropy condition and channel number on the subjective mean clarity rating** | | | | | | |
| --- | --- | --- | --- | --- | --- | --- |
| contrast | channel_nr | estimate | SE | df | t.ratio | p.value |
| el - em | 12 | 0.67 | 0.66 | 54 | 1.01 | 0.74 |
| el - eh | 12 | 4.16 | 0.93 | 54 | 4.45 | 0.00 |
| el - elmm | 12 | 19.45 | 1.86 | 54 | 10.43 | 0.00 |
| em - eh | 12 | 3.49 | 0.96 | 54 | 3.64 | 0.00 |
| em - elmm | 12 | 18.78 | 1.82 | 54 | 10.31 | 0.00 |
| eh - elmm | 12 | 15.29 | 2.03 | 54 | 7.55 | 0.00 |
| el - em | 6 | 3.03 | 0.81 | 54 | 3.72 | 0.00 |
| el - eh | 6 | 13.66 | 1.51 | 54 | 9.02 | 0.00 |
| el - elmm | 6 | 30.78 | 1.67 | 54 | 18.42 | 0.00 |
| em - eh | 6 | 10.63 | 1.32 | 54 | 8.07 | 0.00 |
| em - elmm | 6 | 27.75 | 1.43 | 54 | 19.39 | 0.00 |
| eh - elmm | 6 | 17.11 | 1.63 | 54 | 10.53 | 0.00 |
| el - em | 3 | 5.23 | 1.34 | 54 | 3.91 | 0.00 |
| el - eh | 3 | 22.63 | 1.51 | 54 | 15.01 | 0.00 |
| el - elmm | 3 | 30.45 | 1.95 | 54 | 15.58 | 0.00 |
| em - eh | 3 | 17.40 | 1.68 | 54 | 10.35 | 0.00 |
| em - elmm | 3 | 25.22 | 2.03 | 54 | 12.44 | 0.00 |
| eh - elmm | 3 | 7.82 | 1.52 | 54 | 5.13 | 0.00 |
| el - em | 1 | 1.30 | 0.58 | 54 | 2.25 | 0.12 |
| el - eh | 1 | 4.25 | 0.63 | 54 | 6.71 | 0.00 |
| el - elmm | 1 | 2.62 | 0.77 | 54 | 3.39 | 0.01 |
| em - eh | 1 | 2.94 | 0.50 | 54 | 5.85 | 0.00 |
| em - elmm | 1 | 1.32 | 0.59 | 54 | 2.24 | 0.13 |
| eh - elmm | 1 | -1.63 | 0.53 | 54 | -3.09 | 0.02 |

**Figure S6.** Scatter plots between subclinical and cognitive trait markers and subjective clarity ratings. P-Values are uncorrected.

R: Pearson Correlation Coefficient; SPQ: Schizotypal Personality Questionnaire^9,10^; AQ: Autism Spectrum Quotient^11^; VCI: Verbal Comprehension Index derived from the WAIS-IV^12^

Confidence rating

We performed a two-factor repeated measure ANOVA to examine the effect of the entropy channel manipulation on mean subjective confidence ratings. Mauchly’s test indicated that the assumption of sphericity had been violated for the main effects of condition (W = 0.39, p < .001, ε = .64) and channel number (W = 0.17, p < .001, ε = .49) and for the interaction effect of condition and channel number (W = 0.03, p < .001, ε = .63). Consequently, degrees of freedom were corrected using Greenhouse-Geisser estimates of sphericity. All effects reported are significant at p < .05. There was a significant main effect of entropy condition, channel number as well as a significant interaction effect of condition and channel on the mean confidence rating (see **Table S5**). Tukey post hoc tests of the significant interaction indicated that for the channel numbers 12, 6, and 3, the mean confidence ratings significantly decreased from low entropy to medium entropy to high entropy sentences, with responses in the low entropy mismatch condition being perceived as the least confident (see **Table S6**). In the 12 channel condition, there was no significant difference in the mean confidence rating between low and medium entropy sentences but participants were more confident in both these conditions compared to high and low entropy (mismatch) sentences. In the 1 channel condition, participants were more confident about the response after a low entropy sentence compared to a medium, high and low entropy mismatch sentence. After medium entropy sentences, participants were more confident than after high entropy sentences, however there was no difference in the mean confidence rating between medium and low entropy mismatch sentence. Finally, participants were significantly more confident after low entropy mismatch sentences in the 1 channel condition than after high entropy sentences.

Pearson correlation analysis between subjective clarity ratings and subclinical and cognitive trait markers are depicted in **Figure S7**.

| **Table S5: Results of the repeated measures ANOVA investigating the effect of condition and channel number on the mean confidence rating** | | | | | |
| --- | --- | --- | --- | --- | --- |
| **Effect** | **df** | **MSE** | **F** | **ges** | **p.value** |
| condition | 1.92, 90.14 | 193.98 | 245.08 *** | .360 | <.001 |
| channel_nr | 1.48, 69.75 | 444.73 | 598.96 *** | .709 | <.001 |
| condition:channel_nr | 5.66, 265.83 | 113.07 | 42.30 *** | .143 | <.001 |

| **Table S6: Results of Tukey post hoc tests investigating the interaction effect of entropy condition and channel number on the mean subjective confidence rating** | | | | | | |
| --- | --- | --- | --- | --- | --- | --- |
| **contrast** | **channel number** | **estimate** | **SE** | **df** | **t-ratio** | **p-value** |
| el - em | 12 | 0.73 | 0.57 | 47 | 1.28 | 0.58 |
| el - eh | 12 | 4.93 | 0.75 | 47 | 6.59 | <.001 |
| el - elmm | 12 | 21.01 | 1.98 | 47 | 10.59 | <.001 |
| em - eh | 12 | 4.21 | 0.79 | 47 | 5.31 | <.001 |
| em - elmm | 12 | 20.29 | 1.86 | 47 | 10.90 | <.001 |
| eh - elmm | 12 | 16.08 | 2.05 | 47 | 7.86 | <.001 |
| el - em | 6 | 2.43 | 0.73 | 47 | 3.34 | 0.01 |
| el - eh | 6 | 12.52 | 1.52 | 47 | 8.23 | <.001 |
| el - elmm | 6 | 35.88 | 2.23 | 47 | 16.08 | <.001 |
| em - eh | 6 | 10.09 | 1.29 | 47 | 7.83 | <.001 |
| em - elmm | 6 | 33.45 | 2.00 | 47 | 16.74 | <.001 |
| eh - elmm | 6 | 23.37 | 2.05 | 47 | 11.40 | <.001 |
| el - em | 3 | 8.92 | 1.51 | 47 | 5.92 | <.001 |
| el - eh | 3 | 29.28 | 1.70 | 47 | 17.27 | <.001 |
| el - elmm | 3 | 42.34 | 2.09 | 47 | 20.22 | <.001 |
| em - eh | 3 | 20.36 | 2.08 | 47 | 9.80 | <.001 |
| em - elmm | 3 | 33.42 | 2.41 | 47 | 13.89 | <.001 |
| eh - elmm | 3 | 13.06 | 1.72 | 47 | 7.60 | <.001 |
| el - em | 1 | 10.31 | 1.69 | 47 | 6.09 | <.001 |
| el - eh | 1 | 22.62 | 2.56 | 47 | 8.85 | <.001 |
| el - elmm | 1 | 13.74 | 2.61 | 47 | 5.27 | <.001 |
| em - eh | 1 | 12.32 | 1.99 | 47 | 6.20 | <.001 |
| em - elmm | 1 | 3.44 | 2.20 | 47 | 1.56 | 0.41 |
| eh - elmm | 1 | -8.88 | 2.43 | 47 | -3.65 | <.001 |

**Figure S7.** Scatter plots between subclinical and cognitive trait markers and subjective confidence ratings. P-Values are uncorrected.

R: Pearson Correlation Coefficient; SPQ: Schizotypal Personality Questionnaire^9,10^; AQ: Autism Spectrum Quotient^11^; VCI: Verbal Comprehension Index derived from the WAIS-IV^12^

**Statistical results showing the impact of sentence entropy behavioural response types**

To investigate the effect of entropy condition on the behavioural response types, repeated-measures ANOVAS were conducted separately for correct responses, no responses, and task-based hallucinations. For the proportion of correct responses, Mauchly’s test indicated that the assumption of sphericity had been violated (W = 0.65, p < .001, ε = .80). Therefore, degrees of freedom were corrected using Greenhouse-Geisser estimates of sphericity. There was a significant main effect of entropy condition (F(2.39, 129.24) = 553.00, p<0.001, general effect size = .852) on the proportion of correct responses. Tukey post hoc tests revealed a graded effect of the entropy condition on the proportion of correct responses with the highest proportion of correct responses in the low entropy condition, followed by the medium and high entropy condition, with the low entropy mismatch condition showing the lowest proportion (see **Table S7**). Pearson correlation analyses between the correct responses and subclinical and cognitive trait markers are depicted in **Figure S8**.

| **Table S7: Results of Tukey post hoc tests investigating the effect of entropy condition on the proportion of correct responses** | | | | | |
| --- | --- | --- | --- | --- | --- |
| contrast | estimate | SE | df | t-ratio | p-value |
| el - em | 8.70 | 0.76 | 54 | 11.51 | <.001 |
| el - eh | 26.95 | 0.95 | 54 | 28.29 | <.001 |
| el - elmm | 40.05 | 1.30 | 54 | 30.81 | <.001 |
| em - eh | 18.25 | 1.05 | 54 | 17.33 | <.001 |
| em - elmm | 31.34 | 1.22 | 54 | 25.64 | <.001 |
| eh - elmm | 13.09 | 1.13 | 54 | 11.63 | <.001 |

**Figure S8.** Scatter plots between subclinical and cognitive trait markers and correct responses. P-Values are uncorrected.

R: Pearson Correlation Coefficient; SPQ: Schizotypal Personality Questionnaire^9,10^; AQ: Autism Spectrum Quotient^11^; VCI: Verbal Comprehension Index derived from the WAIS-IV^12^

For the proportion of no responses, Mauchly’s test indicated that the assumption of sphericity had been violated (W = 0.44, p < .001, ε = .75). Therefore, degrees of freedom were corrected using Greenhouse-Geisser estimates of sphericity. There was a significant main effect of entropy condition (F(2.25, 121.29)=64.98, p<0.001, eta=0.199) on the proportion of no responses. Tukey post hoc tests revealed a graded effect of the entropy condition on the proportion of no responses with the highest proportion of no responses in the high entropy condition, followed by the low entropy mismatch and medium entropy condition, with the low entropy condition showing the lowest proportion (see **Table S8**). Pearson correlation analyses between the no responses and subclinical and cognitive trait markers are depicted in **Figure S9**.

| **Table S8: Results of Tukey post hoc tests investigating the effect of entropy condition on the proportion of no responses** | | | | | |
| --- | --- | --- | --- | --- | --- |
| contrast | estimate | SE | df | t.ratio | p.value |
| el - em | -3.75 | 0.63 | 54 | -5.92 | <.001 |
| el - eh | -13.55 | 1.24 | 54 | -10.95 | <.001 |
| el - elmm | -8.50 | 1.19 | 54 | -7.13 | <.001 |
| em - eh | -9.80 | 1.04 | 54 | -9.38 | <.001 |
| em - elmm | -4.75 | 0.87 | 54 | -5.49 | <.001 |
| eh - elmm | 5.05 | 1.08 | 54 | 4.65 | <.001 |

**Figure S9.** Scatter plots between subclinical and cognitive trait markers and no responses. P-Values are uncorrected.

R: Pearson Correlation Coefficient; SPQ: Schizotypal Personality Questionnaire^9,10^; AQ: Autism Spectrum Quotient^11^; VCI: Verbal Comprehension Index derived from the WAIS-IV^12^

For the proportion of task-based hallucinations, Mauchly’s test indicated that the assumption of sphericity had been violated (W = 0.30, p < .001, ε = .63). Therefore, degrees of freedom were corrected using Greenhouse-Geisser estimates of sphericity. There was a significant main effect of entropy condition (F(1.89, 102.05) = 199.22, p<0.001, general effect size = 0.617) on the proportion of no responses. Tukey post hoc tests revealed a graded effect of the entropy condition on the proportion of task-based hallucinations with the highest proportion of task-based hallucinations in the low entropy mismatch condition, followed by the high and medium entropy condition, with the low entropy condition showing the lowest proportion (see **Table S9**). Pearson correlation analyses between the no responses and subclinical and cognitive trait markers are depicted in **Figure S10**.

| **Table S9: Results of Tukey post hoc tests investigating the effect of entropy condition on the proportion of task-based hallucinations** | | | | | |
| --- | --- | --- | --- | --- | --- |
| contrast | estimate | SE | df | t-ratio | p-value |
| el - em | -4.95 | 0.67 | 54 | -7.35 | <.001 |
| el - eh | -13.41 | 1.42 | 54 | -9.47 | <.001 |
| el - elmm | -31.55 | 1.83 | 54 | -17.23 | <.001 |
| em - eh | -8.45 | 1.20 | 54 | -7.04 | <.001 |
| em - elmm | -26.59 | 1.57 | 54 | -16.92 | <.001 |
| eh - elmm | -18.14 | 1.36 | 54 | -13.31 | <.001 |

**Figure S10.** Scatter plots between subclinical and cognitive trait markers and task-based hallucinations. P-Values are uncorrected.

R: Pearson Correlation Coefficient; SPQ: Schizotypal Personality Questionnaire^9,10^; AQ: Autism Spectrum Quotient^11^; VCI: Verbal Comprehension Index derived from the WAIS-IV^12^

**Computational modelling – Quality checks**

**Figure S11:** Trace plots for direct-effect model with individual effects for 55 participants for each population gamma showing successful convergence of four Markov Chains, each with 2000 iterations (warmup=1000; thin=1; post-warmup draws per chain=1000, total post-warmup draws=4000 for each gamma).

**Figure S12**: Pairwise scatterplots and marginal posterior distributions of population gammas. Diagonal histograms indicate normal distributions for all gammas. Scatterplots indicate little to no correlations between gammas.

**P600 Control Analysis**

After visual inspection of the ERPs, we performed linear mixed models (LMMs) separately for the subclinical and cognitive trait markers to investigate the effect of the trait markers and with sentence predictability levels during a later time frame (600 to 900ms post final-word onset). Sentence predictability level (high, medium, low, high mismatch), subclinical (SPQ/AQ) and cognitive (VCI) trait markers, and their interactions were included as fixed effects, while the individual participant and 15 centro-parietal electrode positions (C3, C1, Cz, C2, C4, CP3, CP1, CPz, CP2, CP4, P3, P1, Pz, P2, P4) were included as random intercepts. The results are depicted in **Table S9**.

For all three LMMs, there was a significant main effect of sentence predictability level. Paired Tukey corrected post-hoc comparison revealed a similar graded effect of sentence predictability level as in the N400 analyses with a more negative amplitude in the high mismatch compared to the in the low predictability condition (SPQ/AQ/VCI: -0.99, 95% CI=[-1.21, -0.76]); a more negative N400 amplitude in the low compared to the medium predictability condition (SPQ/AQ/VCI: -0.95, 95% CI=[-1.17, -0.72]). In contrast to the results in the N400 time-window, there was no significant amplitude difference between the medium and the high predictability condition (SPQ/AQ/VCI:-0.05, 95% CI=[-0.28, 0.18]).

In contrast to the N400 time-window, increasing SPQ did not have a significant effect in the high predictability baseline condition. We found a significant interaction effect between SPQ and the medium and high mismatch condition compared to the effect of SPQ in the baseline condition, indicating that the mean amplitude difference between the medium and the high predictability condition and the mean amplitude difference between the high mismatch and the high predictability condition increased with increasing SPQ. Follow-up simple slope analyses did not reveal a significant change of the mean amplitude with increasing SPQ in any condition on their own (high: 0.02, SE=0.23, t=0.09, *p*=0.933; medium: -0.21, SE=0.23, t=-0.92, *p*=0.359; low:-0.09, SE=0.23, t=-0.39, *p*=0.698; high mismatch: -0.33, SE=0.23, t=-1.43, *p*=0.157).

For the LMM including AQ, there was a significant interaction effect between AQ and the high mismatch condition compared to the effect of AQ in the baseline condition, suggesting that the mean amplitude difference between the high and high mismatch condition increased with increasing AQ. Follow-up simple slope analyses did not reveal a significant change of the mean amplitude with increasing AQ in any condition on their own (high: 0.05, SE=0.23, t=0.23, *p*=0.819; medium: 0.09, SE=0.23, t=0.39, *p*=0.696; low: 0.20, SE=0.23, t=0.57, *p*=.572; high mismatch: 0.00, SE=0.23, t=0.00, *p*=0.999).

For the LMM including VCI, there was a significant interaction effect between VCI and the high mismatch condition compared to the effect of VCI in the baseline condition, indicating an increasing mean amplitude difference between the high and high mismatch condition with increasing VCI. Follow-up simple slope analyses did not reveal a significant change of the mean N400 amplitude with increasing VCI in any condition on their own (high: 0.25, SE=0.23, t=1.12, *p*=0.267; medium: 0.41, SE=0.23, t=1.80, *p*=0.077; low: 0.27, SE=0.23, t=1.21, *p*=0.230; high mismatch: -0.17, SE=0.23, t=-0.51, *p*=0.610).

| **Table S9: P600 LMM Results** | | | | | | | | | |
| --- | --- | --- | --- | --- | --- | --- | --- | --- | --- |
|  | **SPQ** | | | **AQ** | | | **VCI** | | |
| *Predictors* | *Estimates* | *CI (95%)* | *P-Value* | *Estimates* | *CI (95%)* | *P-Value* | *Estimates* | *CI (95%)* | *P-Value* |
| Intercept | 3.65 | 2.52 –  4.78 | **<0.001** | 3.65 | 2.52 –  4.78 | **<0.001** | 3.65 | 2.52 –  4.78 | **<0.001** |
| Medium Entropy | -0.05 | -0.22 –  0.12 | 0.570 | -0.05 | -0.22 –  0.12 | 0.570 | -0.05 | -0.22 –  0.12 | 0.568 |
| High Entropy | -1.00 | -1.17 –  -0.82 | **<0.001** | -1.00 | -1.17 –  -0.82 | **<0.001** | -1.00 | -1.17 –  -0.82 | **<0.001** |
| Low Entropy (Mismatch) | -1.98 | -2.16 –  -1.81 | **<0.001** | -1.98 | -2.16 –  -1.81 | **<0.001** | -1.98 | -2.16 –  -1.81 | **<0.001** |
| Trait Marker | 0.02 | -0.43 –  0.46 | 0.932 | 0.05 | -0.39 –  0.50 | 0.818 | 0.25 | -0.19 –  0.70 | 0.263 |
| Trait Marker: Medium Entropy | -0.23 | -0.40 –  -0.06 | **0.009** | 0.04 | -0.14 –  0.21 | 0.674 | 0.15 | -0.02 –  0.32 | 0.081 |
| Trait Marker: High Entropy | -0.11 | -0.28 –  0.06 | 0.221 | 0.08 | -0.10 –  0.25 | 0.383 | 0.02 | -0.15 –  0.19 | 0.813 |
| Trait Marker: Low Entropy (Mismatch) | -0.34 | -0.52 –  -0.17 | **<0.001** | -0.05 | -0.22 –  0.12 | 0.554 | -0.37 | -0.54 – -0.20 | **<0.001** |
| **Random Effects** | | | | | | | | | |
| σ^2^ | 3.18 | | | 3.19 | | | 3.16 | | |
| τ_00_ | 2.61 _participant_ | | | 2.63 _participant_ | | | 2.59 _participant_ | | |
|  | 4.20 _electrodeposition_ | | | 4.20 _electrodeposition_ | | | 4.19 _electrodeposition_ | | |
| ICC | 0.68 | | | 0.68 | | | 0.68 | | |
| N | 15 _electrodeposition_ | | | 15 _electrodeposition_ | | | 15 _electrodeposition_ | | |
|  | 55 _participant_ | | | 55 _participant_ | | | 55 _participant_ | | |
| Observations | 3300 | | | 3300 | | | 3300 | | |
| Marginal R^2^ / Conditional R^2^ | 0.065 / 0.702 | | | 0.062 / 0.701 | | | 0.069 / 0.704 | | |

**References**

1.	Peirce, J. *et al.* PsychoPy2: Experiments in behavior made easy. *Behav. Res. Methods* **51**, 195–203 (2019).

2.	R Core Team. *R: A Language and Environment for Statistical Computing*. (R Foundation for Statistical Computing, Vienna, Austria, 2020).

3.	Wickham, H. *Ggplot2: Elegant Graphics for Data Analysis*. (Springer-Verlag New York, 2016).

4.	Lüdecke, D. *sjPlot: Data Visualization for Statistics in Social Science*. (2024).

5.	Bates, D., Mächler, M., Bolker, B. & Walker, S. Fitting Linear Mixed-Effects Models Using **lme4**. *J. Stat. Softw.* **67**, (2015).

6.	Lenth, R. V. *Emmeans: Estimated Marginal Means, Aka Least-Squares Means*. (2020).

7.	Long, J. A. interactions: Comprehensive, User-Friendly Toolkit for Probing Interactions. 1.2.0 https://doi.org/10.32614/CRAN.package.interactions (2019).

8.	Green, P. & MacLeod, C. J. SIMR : an R package for power analysis of generalized linear mixed models by simulation. *Methods Ecol. Evol.* **7**, 493–498 (2016).

9.	Raine, A. The SPQ: A Scale for the Assessment of Schizotypal Personality Based on DSM-III-R Criteria. *Schizophr. Bull.* **17**, 555–564 (1991).

10.	Wuthrich, V. & Bates, T. C. Confirmatory Factor Analysis of the Three-Factor Structure of the Schizotypal Personality Questionnaire and Chapman Schizotypy Scales. *J. Pers. Assess.* **87**, 292–304 (2006).

11.	Baron-Cohen, S., Wheelwright, S., Skinner, R., Martin, J. & Clubley, E. The Autism-Spectrum Quotient (AQ): Evidence from Asperger Syndrome/High-Functioning Autism, Malesand Females, Scientists and Mathematicians. *J. Autism Dev. Disord.* **31**, 5–17 (2001).

12.	Wechsler, D. Wechsler Adult Intelligence Scale--Fourth Edition. https://doi.org/10.1037/t15169-000 (2012).
